# Fast, Nondestructive, and Broadband Dielectric Characterization for Polymer Sheets

**DOI:** 10.3390/polym12091891

**Published:** 2020-08-21

**Authors:** Hsin-Yu Yao, Dan-Ru Hsiao, Tsun-Hsu Chang

**Affiliations:** Department of Physics, National Tsing Hua University, 101 Section 2 Kuang Fu Road, Hsinchu 300, Taiwan; s5te633v@hotmail.com (H.-Y.Y.); goo@gapp.nthu.edu.tw (D.-R.H.)

**Keywords:** polymer dielectric characterization, nearfield measurement, mode converter, transmission/reflection method

## Abstract

We propose a compact nearfield scheme for fast and broadband dielectric characterization in the microwave region. An open-type circular probe operated in the high-purity TE_01_ mode was developed, showing a strongly confined fringing field at the open end. This fringing field directly probed the freestanding sheet sample, and the overall reflection was measured. Without sample-loading processes, both of the system assembling time and the risk of sample damage can be significantly reduced. In addition, the nearfield measurement substantially simplifies the calibration and the retrieval theory, facilitating the development of easy-to-integrate and easy-to-calibrate dielectric characterization technique. The dielectric properties of more than ten polymers were characterized from 30 GHz to 40 GHz. We believe that this work fulfills the requirement of the fast diagnostic in the industrial manufactures and also provides valuable high-frequency dielectric information for the designs of 5G devices.

## 1. Introduction

With the advent of the 5th generation mobile networks (5G), numerous high-frequency circuits, such as power amplifiers [1,2], flexible antennas [3], and low-pass filters [4], are required for connecting billions of mobile devices and sharing a massive amount of data. To improve the performance and the integration of these microdevices, reducing the signal loss within the circuits and inhabiting the leakage current in the transistors become important tasks. For designing and optimizing, it is essential to investigate the broadband dielectric properties of the circuit-board materials. On the other hand, the nanocomposite field has grown quickly in recent years [5,6,7,8]. By doping dielectric/magnetic nanoparticles into polymer matrices, the electromagnetic properties of the composites can be precisely controlled, realizing many terahertz (THz) and optical devices such as multilayer anti-reflection coatings [9], and all-dielectric waveguides [10]. To understand the electric functionality of the nanocomposites, fast and broadband dielectric characterization techniques at the microwave to the terahertz regimes are also required. Over the past decades, various methods have been developed to meet this goal from 1 GHz to 1 THz [11,12,13,14,15,16,17,18,19,20].

The conventional cavity methods, utilizing the field enhancement of the resonant nature, are able to characterize the materials with various permittivity [12,13]. However, those methods are very narrowband owing to the strict resonant conditions. For broadband characterizations, non-resonant techniques that measure the scattering parameters (transmission/reflection) in waveguides were invented [11,14,15,16,17]. Since the samples are enclosed by the waveguides and all experimental components (e.g., adapters and widows) can be well-calibrated, accurate and stable broadband results are expected. Nevertheless, sample preparation for these methods is usually difficult [14,15,16]. On the one hand, machining samples to meet the waveguide geometry is costly and might lead to sample damage. On the other hand, it is time-consuming to integrate the measurement setup due to the processes of sample loading, position/orientation adjustments, and the avoidance of the contact gaps [17].

The quasi-optical technique in the microwave region [18,19,20], and the time-domain spectroscopy in the terahertz region [21,22,23], can overcome these challenges. It composes of a set of antennas (source and receiver) to apply the far-field measurement in free space [18,19,20]. Unfortunately, the beam diffraction or the employment of multiple focusing devices severely complicate the experimental setups and the calibration procedures. In short, there is an urgent need to develop a compact, easy-to-integrate, and easy-to-calibrate dielectric characterization system.

In this work, we propose an open-ended circular-waveguide probe for fast and broadband dielectric characterization. The probe operated in the circular TE_01_ mode [24,25] is directly attached to a freestanding sheet sample. Since there is no need to load the sample, the sample preparation time, the system assembling time, and the risk of sample damage can be greatly reduced as compared to References [11,12,13,14,15,16,17]. On the other hand, the TE_01_ mode exhibits purely azimuthal surface current, implying very weak diffraction as it radiates from the waveguide to the sample. This unique feature facilitates the nearfield measurement, making the calibration much easier than References [18,19,20]. We developed a semi-analytical model with a reliable single-mode approximation to simplify the dielectric retrieval process. More than ten polymer sheets were tested, and the result shows good agreement with the full-wave simulation results as well as the previously reported data [26,27,28,29,30,31,32,33,34,35,36].

## 2. Scheme for the Fast Dielectric Characterization

**Figure 1:**
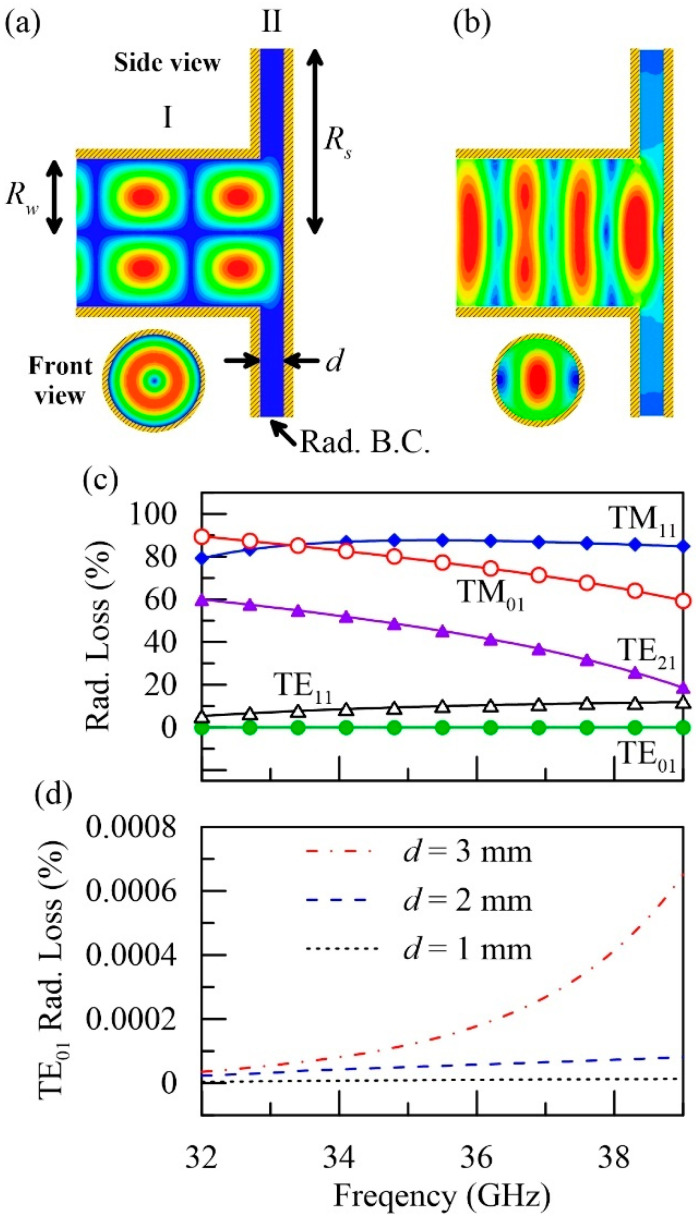
Fringing field patterns of the open-ended circular waveguide probe operated at (**a**) TE_01_ mode and (**b**) TE_11_ mode. (**c**) Radiation loss versus frequency of the first five circular waveguide modes. (**d**) Radiation loss versus frequency of the TE_01_ mode under the three different sample (air) thicknesses.

Simplifying the sample-loading process (necessary for References [11,12,13,14,15,16,17]) is a major challenge for the fast dielectric characterization. Inspired by the quasi-optical techniques [18,19,20], we propose an open-ended configuration delineated in Figure 1a, in which the end of a circular waveguide (Region I) directly probes the front surface of the sample at Region II. The sample with a thickness of d is freestanding in open space, and its rear side is attached to a metal plate for enhancing the total reflection. To avoid the beam diffraction and the derived complexity in the calibrations (suffered by the far-field measurements [18,19,20]), properly choosing the operating waveguide mode is necessary.

Considering the TE_01_ mode in an empty circular $dR_{w}$ waveguide with a radius of $R_{w}$, its electromagnetic (EM) field components are [37],

(1) $$\begin{array}{l} E_{\phi }=\frac{-ik_{0}}{k_{c}}E_{0}J_{0}^{\prime }\left(k_{c}\rho \right)e^{ik_{z0}z-i\omega t}\\ H_{\rho }=\frac{ik_{z0}}{k_{c}}H_{0}J_{0}^{\prime }\left(k_{c}\rho \right)e^{ik_{z0}z-i\omega t}\\ H_{z}=H_{0}J_{0}\left(k_{c}\rho \right)e^{ik_{z}z-i\omega t} \end{array}$$

where ω is the angular frequency of the EM wave, and $E_{0}$ ($H_{0}$) represents the electric-field (magnitude-field) amplitude. $k_{0}=\omega /c$, $k_{c}=0.382/R_{w}$, and $k_{z0}^{2}=k_{0}^{2}-k_{c}^{2}$, respectively, describe the free-space wavenumber, the cutoff wavenumber, and the propagation constant, where c is the speed of light in vacuum. The notation $J_{0}^{\prime }$ refers to the derivative of $J_{0}$ (first-kind Bessel function). All the other field components (not included in Equation (1)) are equal to zero. 

The electric field of the TE_01_ mode is purely azimuthal, driving an azimuthal surface current on the waveguide wall ($\propto \hat{\rho }\times \vec{H}$). Such azimuthal surface current is insensitive to the radial geometric perturbations [37], such as the slotted waveguide structures [38], and the open-ended junction proposed here. This special feature guarantees that the fringing field in Region II can still preserve the same field pattern and the dispersion of the TE_01_ mode, propagating over a reasonably long distance in the sample. In other words, the beam diffraction can be significantly suppressed.

For verification, we used HFSS (High-Frequency Structure Simulator, ANSYS, Canonsburg, RA, USA) to simulate the steady-state field distributions (with $R_{w}=6.02\;\mathrm{mm}$, $R_{s}=15\;\mathrm{mm}$, and d=2 mm). The side edges of Region II (at $\rho =R_{s}$) were set as the radiation boundaries to save the computation time. The result of the TE_01_ incidence is demonstrated in Figure 1a, while that for the TE_11_ incidence (i.e., the fundamental circular waveguide mode) is shown in Figure 1b for comparison. The fringing field of the TE_01_ (TE_11_) mode in Region II is highly (weakly) confined, indicating very weak (strong) diffraction. The radiation losses (defined by $1-|R{|}^{2}$; R is the total field reflection coefficient) of the first five lower-order modes (TE_11_, TM_01_, TE_21_, TM_11_, and TE_01_) are shown in Figure 1c. The loss of the TE_01_ mode is negligible, corresponding to the highly confined fringing field, as shown in Figure 1a. On the contrary, the radiation losses of the other modes are all higher than 5%, owing to their highly diffracted feature (e.g., Figure 1b for TE_11_). The TE_01_ radiation losses under three different thicknesses (d=1, 2, and 3 mm) are illustrated in Figure 1d. As shown, the loss remains low with the increase of the free-propagating length. Consequently, the TE_01_ mode is the best candidate for nearfield reflection measurement. This scheme combines the advantages of the waveguide systems [11,12,13,14,15,16,17] (highly confined field and easy-to-calibrate), and the advantage of the quasi-optical systems [18,19,20] (simple setup and easy-to-integrate), presenting the possibility for fast, efficient, and broadband dielectric characterization.

## 3. Theoretical Model for the Complex Permittivity Retrieval

A concise theory is developed for retrieving sample’s complex permittivity from the measured reflection coefficient. When the TE_01_ mode impinges on the open end (z=0), it will be partially reflected (as the ballistic reflection) and partially radiated into the sample. The radiation propagates over the sample and then is reflected by the metal plate (z=d). The reflected signal subsequently bounces back and forth inside the sample, forming a sequence of reflection echoes. The steady-state total reflection is the superposition of the ballistic reflection and the all following echoes.

**Figure 2:**
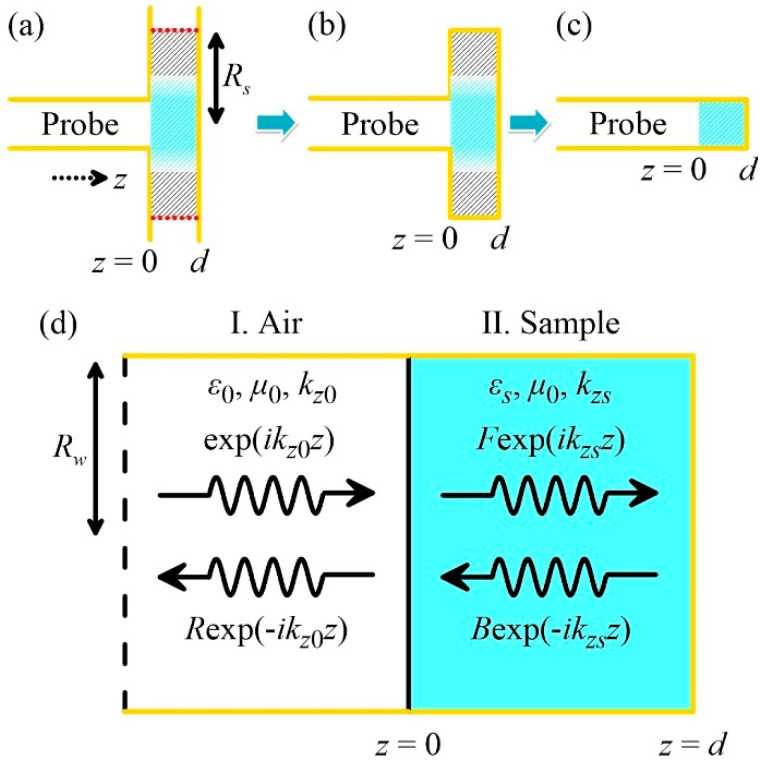
Simplification procedure of the theoretical retrieval model. (**a**) Original open-ended scheme. Red dotted lines indicate the radiation boundaries on the side edges. (**b**) First-step simplification: a closed system with the geometric change in the waveguide radius. The radiation boundaries in (**a**) are replaced by the metals. (**c**) Second-step simplification: a closed and uniform waveguide system. (**d**) Magnified diagram of (**c**) with the details for the single-mode approximation.

In general, the complexity of the coupling between a waveguide mode and the open-space diffracted modes (i.e., the plane waves propagating toward different directions) is very high. By taking advantage of the highly confined TE_01_ fringing field in open space, it is possible to simplify the model. The original scheme is illustrated in Figure 2a, in which the sample cross-section is assumed to be much larger than the wavelength ($R_{s}>>\lambda$). Since the fringing field can never reach the sample edge (highlighted by the red dashes in Figure 2a), it is reasonable to replace the radiation boundaries by the metallic boundaries. The original “open system” thus becomes a “closed system” (Figure 2b). However, as the waveguide geometry in Figure 2b is discontinuous at z=0, multiple higher-order TE_0*m*_ modes must be excited at the junction, jointly with the TE_01_ mode to satisfy the boundary conditions. This process is known as the modal effect [37]. 

Dealing with the modal analysis [10,17,39] is not the main focus of this work; instead, we further simplify the model according to Figure 2c. Owing to the strong field confinement of the TE_01_ radiation (as demonstrated in Figure 1a), Figure 2b is approximately equivalent to a uniform and closed-ended waveguide with the under-test sample locating at its terminal. We can therefore adopt the single-mode approximation (with only TE_01_) to solve the overall reflection. It is worth emphasizing that this single-mode approximation is not valid for other operating modes that exhibit highly diffracted fringing fields in open space (e.g., the lower-order modes listed in Figure 1c).

Based on this simplified configuration, the steady-state fields in each region are shown in Figure 2d. The transverse field components in the empty waveguide (Region I) can be expressed as

(2) $$\begin{array}{l} E_{\phi }^{I}=\frac{-i\omega \mu_{0}}{k_{c}}J_{0}^{\prime }\left(k_{c}\rho \right)\left(e^{ik_{z0}z}+Re^{-ik_{z0}z}\right)e^{-i\omega t}\\ H_{\rho }^{I}=\frac{ik_{z0}}{k_{c}}J_{0}^{\prime }\left(k_{c}\rho \right)\left(e^{ik_{z0}z}-Re^{-ik_{z0}z}\right)e^{-i\omega t} \end{array}$$

where R represents the overall field reflection coefficient. The transverse field components in the sample region (with permittivity $\varepsilon_{s}$ and permeability $\mu_{0}$) are

(3) $$\begin{array}{l} E_{\phi }^{\mathrm{II}}=\frac{-i\omega \mu_{0}}{k_{c}}J_{0}^{\prime }\left(k_{c}\rho \right)\left(Fe^{ik_{zs}z}+Be^{-ik_{zs}z}\right)e^{-i\omega t}\\ H_{\rho }^{\mathrm{II}}=\frac{ik_{zs}}{k_{c}}J_{0}^{\prime }\left(k_{c}\rho \right)\left(Fe^{ik_{zs}z}-Be^{-ik_{zs}z}\right)e^{-i\omega t} \end{array}$$

in which F (B) denotes the forward-wave (backward-wave) coefficient and $k_{zs}^{2}=\mu_{0}\varepsilon_{s}\omega^{2}-k_{c}^{2}$. Applying the boundary conditions at z=0 that require $E_{\phi }^{I}{|}_{z=0}=E_{\phi }^{\mathrm{II}}{|}_{z=0}$ and $H_{\rho }^{I}{|}_{z=0}=H_{\rho }^{\mathrm{II}}{|}_{z=0}$ and the boundary condition at z=L that forces $E_{\phi }^{\mathrm{II}}{|}_{z=L}=0$ due to the metallic reflector, we obtain

(4) $$R=\frac{-\left(k_{z0}-k_{zs}\right)e^{-2ik_{zs}d}+\left(k_{z0}+k_{zs}\right)}{-\left(k_{z0}+k_{zs}\right)e^{-2ik_{zs}d}+\left(k_{z0}-k_{zs}\right)}$$

Although Equation (4) resembles the solution of the two-section configuration with one end closed (Figure 2c), it serves as an approximated solution for the present “open scheme” as long as we operate with the circular TE_01_ modes. Note that Equation (4) is a transcendental equation; numerical root searching is thus required to retrieve the sample’s permittivity $\varepsilon_{s}$ embedded in $k_{zs}$.

## 4. Experimental Setup and Comparison with HFSS Simulation

**Figure 3:**
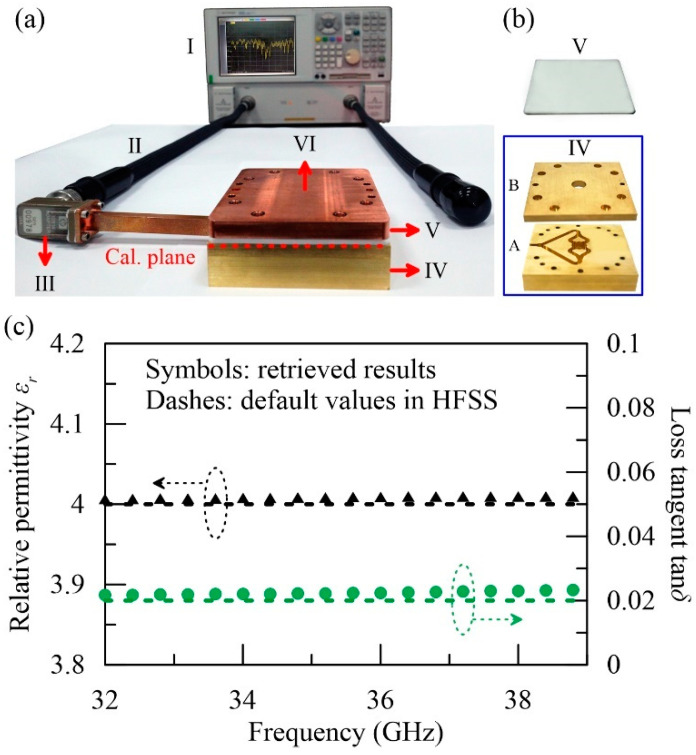
(**a**) Experimental setup. Part I: performance network analyzer. Part II: 2.4 mm flexible coaxial cable. Part III: rectangular TE_10_ mode converter. Part IV: home-made circular TE_01_ mode converter, composed of a power-dividing junction (component A in (**b**)) and a mode-converting tube (component B in (**b**)). Part V: sample under test. Part VI: back metal plate. (**c**) Retrieved results obtained by HFSS simulation. Black triangles (green circles): retrieved $\varepsilon_{r}$ (tanδ) based on the theory proposed in Section 3. The corresponding default values set in HFSS are plotted in dashed curves for comparison.

The photograph of the experimental setup is demonstrated in Figure 3a. Part I is the performance network analyzer (PNA, Agilent E8363B, Agilent Technologies, Santa Clara, CA, USA), which was connected with a 2.4 mm coaxial cable (part II). This cable was attached to an adapter (part III), converting the TEM signal to the TE_10_ mode in the K_a_-band rectangular waveguide and vice versa. For fast dielectric characterization, exciting a high-purity TE_01_ mode in a circular waveguide is essential. Based on our previous works [24,25], a typical Y-type TE_01_ mode converter was designed, fabricated, and connected at part IV, severing as the open-ended circular probe. It comprises two-stage power-dividing junctions made of branched K_a_-band rectangular waveguides (part A in Figure 3b), followed by a mode-converting section made of circular waveguide (part B in Figure 3b). According to the HFSS simulation, the purity of the TE_01_ mode achieves more than 99% ranging from 32 GHz to 39 GHz [10,25] (see Supplementary Materials S1 for its detail characteristics). A sheet sample (part V) is sandwiched between the TE_01_ mode converter (part IV) and a metal plate (part VI). The cross-sectional areas of all the samples are 80 mm × 80 mm, much larger than the probe size with $R_{w}=6.02\;\mathrm{mm}$. The sample thicknesses range between 1 mm and 2 mm. Notice that the reference plane is calibrated to the interface between parts IV and V, as indicated by the red dashed line in Figure 3a (see Supplementary Materials S2 for the detail calibration procedure). The overall reflection was recorded by the PNA and analyzed with Equation (4) to retrieve the complex permittivity.

For verification, we used HFSS to simulate all the above procedures, including the calibration of the mode converter and the permittivity retrieval of an artificial under-test material. The relative permittivity ($\varepsilon_{r}$) of the under-test sample is set as 4, and its loss tangent (tanδ) is set as 0.02 over the whole spectral window. The sample thickness is 1 mm. With the scattering data of the mode converter (see Supplementary Materials S2), we can extract the complex permittivity of the sample under test based on Equation (4). The result is plotted in Figure 3c to compare with the default values. The retrieved $\varepsilon_{r}\sim 4.009$ shows excellent agreement with the default with an error less than 0.5% over the whole spectral window. On the other hand, the retrieved tanδ~0.022 is slightly larger than the default because of the open boundaries of the freestanding sample; however, the accuracy is still acceptable. To further improve the accuracy of tanδ, the modal analysis is required for the less-simplified configuration shown in Figure 2b, i.e., the closed waveguide system with a sharp radius change. 

## 5. Results and Discussions

In the experiment, we characterized eleven polymers in total, including three common plastics (polyethylene (PE), polypropylene (PP), and polymethylmethacrylate (PMMA)), one heat/chemical-resisting plastic (polytetrafluoroethylene (PTFE)), one engineering plastic (polycarbonate (PC)), two piezoelectric materials (polyvinyl chloride (PVC) and polyvinylidene difluoride (PVDF)), and four circuit boards (FR4). Two of the FR4 boards are yellow (FR4_Y_a_ and FR4_Y_b_), and the other two are green (FR4_Y_a_ and FR4_G_b_). The subscripts “a” and “b” stand for the samples provided by different manufacturers. 

**Figure 4:**
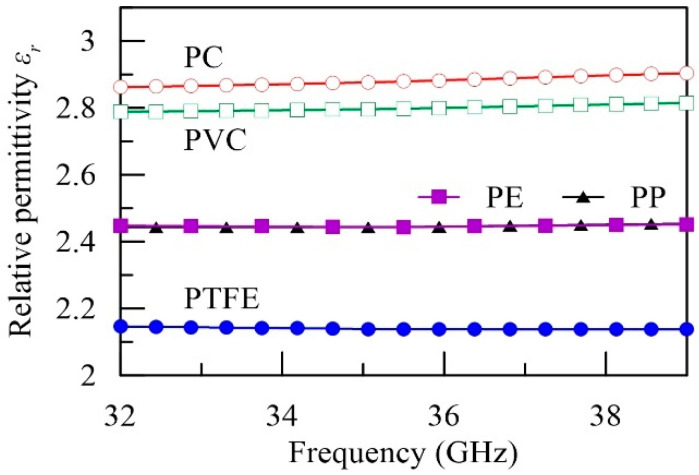
Relative permittivity of the low-loss materials.

For clarity, the retrieved data are classified into two groups: the low-loss and the high-loss groups. The relative permittivity (εr≡Re[εs]/ε0) of the low-loss group is plotted in Figure 4. The loss tangent (tanδ≡Im[εs]/Re[εs]) is not shown, because it is too low to test (less than 10^−3^) by the present scattering scheme. Instead, the conventional cavity methods with strong field enhancement are more suitable [12,13]. Among these low-loss polymers, PTFE exhibits the lowest $\varepsilon_{r}$ of around 2.14; PE and PP, belonging to the polyolefin, show very close $\varepsilon_{r}$ of around 2.45, and PVC and PC have relatively large $\varepsilon_{r}$, ranging from 2.8 to 2.9.

**Figure 5:**
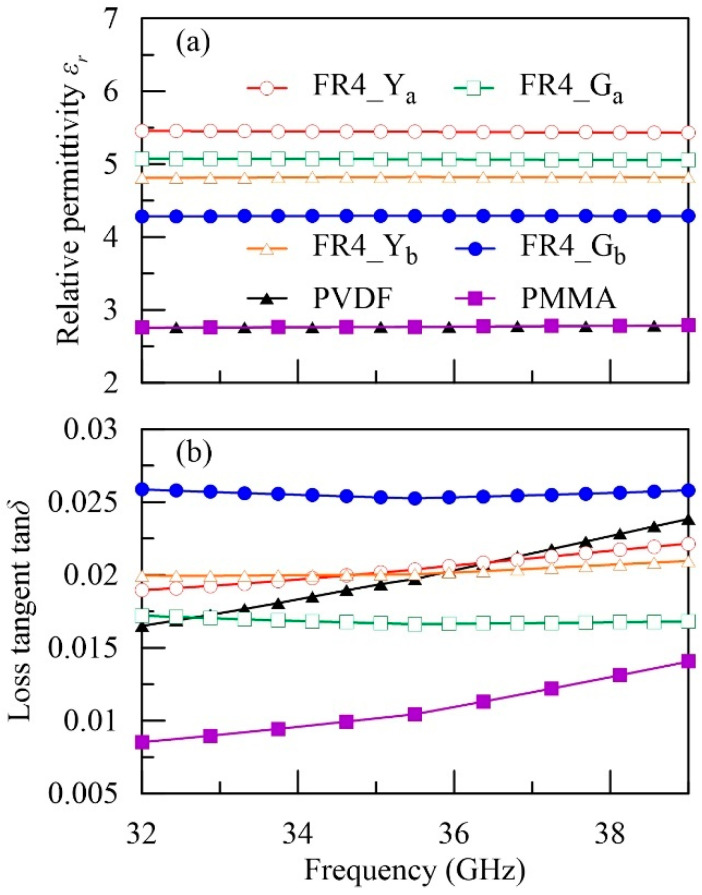
(**a**) Relative permittivity and (**b**) loss tangent of the high-loss materials.

Regarding the high-loss group, $\varepsilon_{r}$ (tanδ) is presented in Figure 5a,b. We found that PMMA and PVDF exhibit quite similar $\varepsilon_{r}$ around 2.77, while PVDF is relatively high-loss, manifested in its larger tanδ around 0.02. On the other hand, the four FR4 boards possess higher and more diverse $\varepsilon_{r}$ (from 4.2–5.5). The higher dielectric constant and the stronger dielectric loss might result from their complex compositions, including epoxy matrices (typical $\varepsilon_{r}\sim 3.5–4$) [17], fiberglass ($\varepsilon_{r}\sim 3–15$) for reinforcement [40], functional fillers, and dyeing materials. Although the concentrations of the latter two are usually low, they can still greatly alter the effective permittivity of the composite due to their high-K nature [41,42]. Besides, our data show that the FR4 boards provided by different manufacturers reveal very different permittivity even if the colors are the same (e.g., FR4_Y_a_ vs. FR4_Y_b_). Such difference must result in very distinct circuit impedances at the high-frequency (5G) regime. This observation manifests the importance of our work, which can easily provide the accurate electrical properties of the targeted boards before any circuit designs. The data at 36 GHz were extracted and summarized in Table 1 with error bars, showing fairly good agreement with the previously reported values (given in the parentheses) [26,27,28,29,30,31,32,33,34,35,36].

## 6. Conclusions

In Summary, this study established a broadband measurement system for fast dielectric characterization. By using the open-ended probe operating in the circular TE_01_ mode, the nearfield measurement of freestanding sheet dielectrics was accomplished. Since the experimental setup is compact, the sample preparation time and the system assembling time were significantly saved, as compared to the conventional cavity methods and the transmission/reflection methods in waveguide systems. The highly confined TE_01_ fringing field also eliminated the complicated calibrations for diffraction, leading to more accurate and stable results. More than 10 polymers (PTFE, PP, PE, PVC, PC, PVDF, PMMA, and FR4) were characterized from 30 GHz to 40 GHz, showing various dielectric constants ranging from 2.1–5.5 and loss tangents ranging from 0.001–0.03. We believe this novel characterization method can benefit the development of the designs, fabrications, and manufacture quality controls of 5G high-frequency devices.

## Figures and Tables

**Table 1:** The dielectric properties of the plastics at 36 GHz.

Material	$\varepsilon_{r}$	tanδ
PTFE	2.14 ± 0.3%	<0.001
	(1.95) [26]	
PP	2.45 ± 0.1%	<0.001
	(2.29~2.30) [27]	
PE	2.44 ± 0.6%	<0.001
	(2.35~2.37) [27]	
PVC	2.8 ± 0.3%	<0.005
	(2.50) [28]	
PC	2.88 ± 0.1%	<0.001
	(2.76) [29]	
PVDF	2.77 ± 0.3%	0.02 ± 11.8%
	(1.5~10) [30,31,32,33]	(0.02~0.16) [30]
PMMA	2.77 ± 0.1%	0.011 ± 16.9%
	(2.60~2.67) [27]	(0.015~0.061) [27]
FR4_G_b_	4.29 ± 0.3%	0.026 ± 4.6%
	(3.5~5) [34,35,36]	(0.015~0.02) [18,19,20]
FR4_Y_b_	4.82 ± 0.2%	0.02 ± 3.1%
FR4_G_a_	5.06 ± 0.2%	0.017 ± 5.2%
FR4_Y_a_	5.44 ± 0.8%	0.02 ± 7.3%

## Supplementary Materials

The following are available online at https://www.mdpi.com/2073-4360/12/9/1891/s1.
