# Supplementary material for: Fast, Nondestructive, and Broadband Dielectric Characterization for Polymer Sheets
Source: Polymers (Basel). 2020 Aug 21;12(9):1891. doi: 10.3390/polym12091891 (PMC7565126; doi:10.3390/polym12091891)
Supplement: Supplementary file 1 [file polymers-12-01891-s001.pdf]

Supplementary materials for "Fast, nondestructive, and broadband dielectric characterization for polymer sheets"

Hsin-Yu Yao, Dan-Ru Hsiao, and Tsun-Hsu Chang*

Department of Physics, National Tsing Hua University, Hsinchu, 30013, Taiwan

S1: Scattering properties of the Y-type TE_{01} mode converter

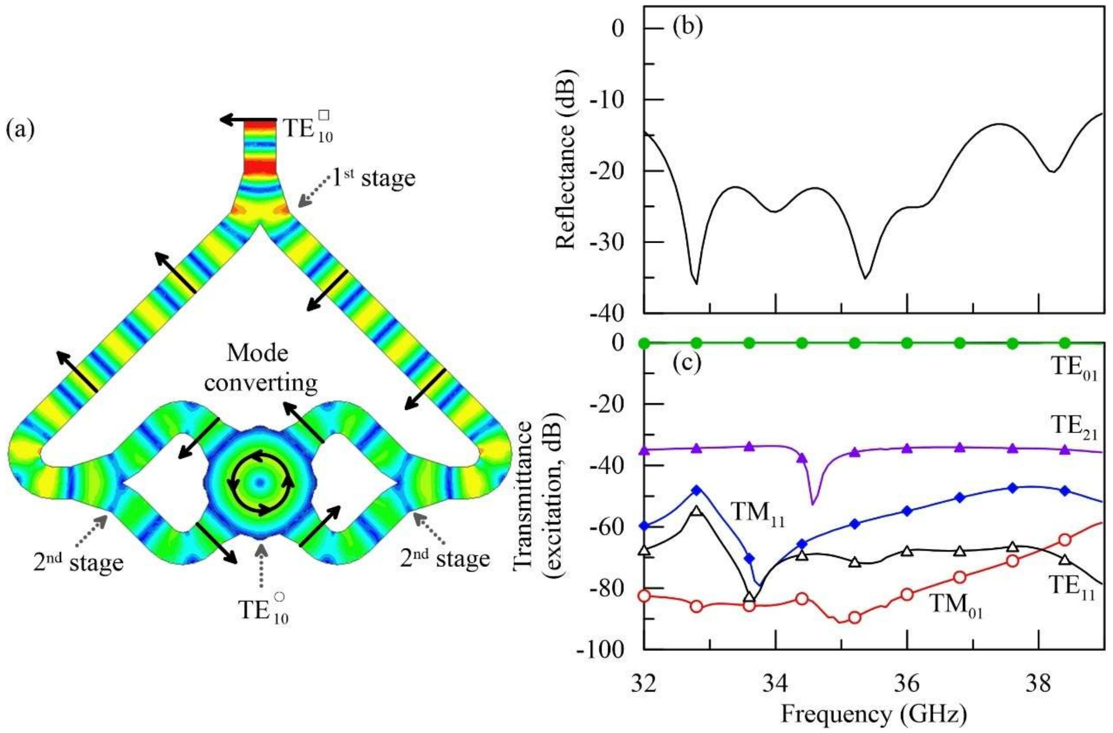

Fig. S1 (a) Electric field distribution of the Y-type TE_{01} mode converter. Black arrows indicate the field polarization. (b) Reflectance of the TE_{10} rectangular waveguide mode. (c) Transmittances (excitations) of the first five circular waveguide modes coupled out by the mode converter.

In this section, we will briefly describe the mechanism of the Y-type mode converter, which is able to couple the TE_{01} circular waveguide mode with high purity. It is composed of two-stage power-dividing junctions made of branched rectangular waveguides and a mode-converting section made of circular waveguide. The electric field profile, with its polarization inside the Y-type TE_{01} mode converter, is plotted in Fig. S1(a).

The input end of the rectangular waveguide is operated in the fundamental TE_{10} mode (denote as $TE_{10}^{\square}$). As the $TE_{10}^{\square}$ injection travels to the 1st-stage power-dividing

junction, it splits into two branches with equal magnitude and phase. The two divisions propagate along their own waveguide tunnels and are further divided into four sub-signals by 2nd-stage power dividers. These four branches are attached symmetrically on the sidewall of the mode-converting circular waveguide. The four linearly-polarized $\mathrm{TE}_{10}^{\square}$ fields simultaneously circumnavigate the circular waveguide to excite the TE circular waveguide modes with four-fold symmetry (*i.e.*, $\mathrm{TE}_{mn}^{\bigcirc}$ with $m = 0, 4, 8$...etc.). This greatly suppresses the excitations of the lower-order modes inside the circular waveguide (*i.e.*, $\mathrm{TE}_{11}^{\bigcirc}$, $\mathrm{TM}_{01}^{\bigcirc}$, $\mathrm{TE}_{21}^{\bigcirc}$, and $\mathrm{TM}_{11}^{\bigcirc}$), owing to the mismatched azimuthal symmetry in the field profile and the polarization. As the operating band is chosen slightly above the $\mathrm{TE}_{01}^{\bigcirc}$ cutoff, only the $\mathrm{TE}_{01}^{\bigcirc}$ mode can be excited and all other allowed higher-order modes ($\mathrm{TE}_{mn}^{\bigcirc}$ with $m = 4, 8$...etc.) are evanescent and thus are also precluded. Consequently, only the very high-purity $\mathrm{TE}_{01}^{\bigcirc}$ mode will emerge at the output end of the mode converter, as shown in Fig. S1(a).

Figures S1(b) and S1(c) shows the scattering spectrums of the mode converter, calculated by HFSS with copper boundary (conductivity is 5.80×10^{7} S/m). A low reflectance of rectangular TE_{10} mode is observed [< -15 dB (3%)], over the major operating band from 32 GHz to 39 GHz. The transmittance of the TE_{01} mode is high [> -0.4 dB (90%)], while the excitations of the other lower-order modes are very low (< -30 dB). This indicates that the purity of the TE_{01} mode achieves more than 99%. The small coupling loss is dominated by the ohmic loss on the copper wall. This high-purity Y-type TE_{01} mode converter serves as the open-ended probe for fast dielectric characterization proposed in the main text.

S2: Three-offset calibration procedure of the TE01 mode converter

To retrieve the exact reflection coefficient of the sample under test [Eq. (4) in the main text], the additional scattering due to the presence of the TE_{01} mode converter should be carefully calibrated. Since the couplings to all other modes are negligible (as demonstrated in S1), such scattering behavior can be simply described by a 2×2 matrix with four elements: S_{11}^{MC} ($\mathrm{TE}_{10}^{\square}\rightarrow\mathrm{TE}_{10}^{\square}$, the reflection from $\square$ back to $\square$), S_{12}^{MC} ($\mathrm{TE}_{01}^{\bigcirc}\rightarrow\mathrm{TE}_{10}^{\square}$, the transmission from $\bigcirc$ to $\square$), S_{21}^{MC} ($\mathrm{TE}_{10}^{\square}\rightarrow\mathrm{TE}_{01}^{\bigcirc}$, the transmission from $\square$ to $\bigcirc$), and S_{22}^{MC} ($\mathrm{TE}_{01}^{\bigcirc}\rightarrow\mathrm{TE}_{01}^{\bigcirc}$, the reflection from $\bigcirc$ back to $\bigcirc$). The symbols $\square$ ($\bigcirc$) denotes the rectangular (circular) waveguide. Note that $S_{12}^{\mathrm{MC}}=S_{21}^{\mathrm{MC}}$ due to the reciprocity, and thus only three elements are independent. To retrieve S_{11}^{MC}, S_{12}^{MC}, and S_{22}^{MC}, we propose to use three offsets for calibration. An offset is a uniform waveguide with the same radius of the converter's output end ($R_w = 6.02$ mm) sealed by a short plane (metal plate) after a thickness of d_j. It is schematically illustrated in Figs. S2(a). We assume the three thicknesses are respectively 0 (*i.e.*, the short scheme in the traditional TRL calibration), d_A, and d_B. Without loss of generality, d_A and d_B are not necessary to match the open condition. The total field reflection coefficient for the calibration scheme shown in Fig. S2(a) is

$$S_{11}^{\mathrm{S}} = S_{11}^{\mathrm{MC}} - \frac{S_{21}^{\mathrm{MC}} S_{12}^{\mathrm{MC}}}{1+S_{22}^{\mathrm{MC}}}, \tag{S1}$$

while the coefficients in the cases with the offset thicknesses d_A and d_B are

$$S_{11}^{\mathrm{OA}} = S_{11}^{\mathrm{MC}} - \frac{S_{21}^{\mathrm{MC}} S_{12}^{\mathrm{MC}} e^{2ik_{z0}d_A}}{1 + S_{22}^{\mathrm{MC}} e^{2ik_{z0}d_A}}, \tag{S2}$$

and

$$S_{11}^{\mathrm{OB}} = S_{11}^{\mathrm{MC}} - \frac{S_{21}^{\mathrm{MC}} S_{12}^{\mathrm{MC}} e^{2ik_{z0}d_B}}{1 + S_{22}^{\mathrm{MC}} e^{2ik_{z0}d_B}}. \tag{S3}$$

The superscripts "S", "OA", and "OB" denote the short, the offset with thickness d_A, and the offset of another thickness d_B, respectively. With some linear algebra, it is easy to represent the two unknowns—S_{11}^{MC} and S_{22}^{MC} in terms of the measurable parameters S_{11}^{S}, S_{11}^{OA}, and S_{11}^{OB}. It yields

$$\begin{bmatrix} S_{11}^{\mathrm{MC}} \\ S_{22}^{\mathrm{MC}} \end{bmatrix} = \begin{bmatrix} -1 + e^{2ik_{z0}d_A} & -\left(S_{11}^{\mathrm{S}} - S_{11}^{\mathrm{OA}}\right) e^{2ik_{z0}d_A} \\ -1 + e^{2ik_{z0}d_B} & -\left(S_{11}^{\mathrm{S}} - S_{11}^{\mathrm{OB}}\right) e^{2ik_{z0}d_B} \end{bmatrix}^{-1} \begin{bmatrix} S_{11}^{\mathrm{S}} e^{2ik_{z0}d_A} - S_{11}^{\mathrm{OA}} \\ S_{11}^{\mathrm{S}} e^{2ik_{z0}d_B} - S_{11}^{\mathrm{OB}} \end{bmatrix}. \tag{S4}$$

The last unknown—S_{12}^{MC} can be further expressed by

$$S_{12}^{\mathrm{MC}} = S_{21}^{\mathrm{MC}} = \sqrt{\left(S_{11}^{\mathrm{MC}} - S_{11}^{\mathrm{S}}\right)\left(1 + S_{22}^{\mathrm{MC}}\right)}. \tag{S5}$$

To validate, we use HFSS to perform the above three-offset calibration procedures. The extracted values are demonstrated in Figs. S2(b)-S2(d) by the red dots. The exact scattering parameters simulated by HFSS are plotted in black solid curves for comparison. As shown, the retrieved data match with the exact values obtained by HFSS, validating the correctness of this calibration method.

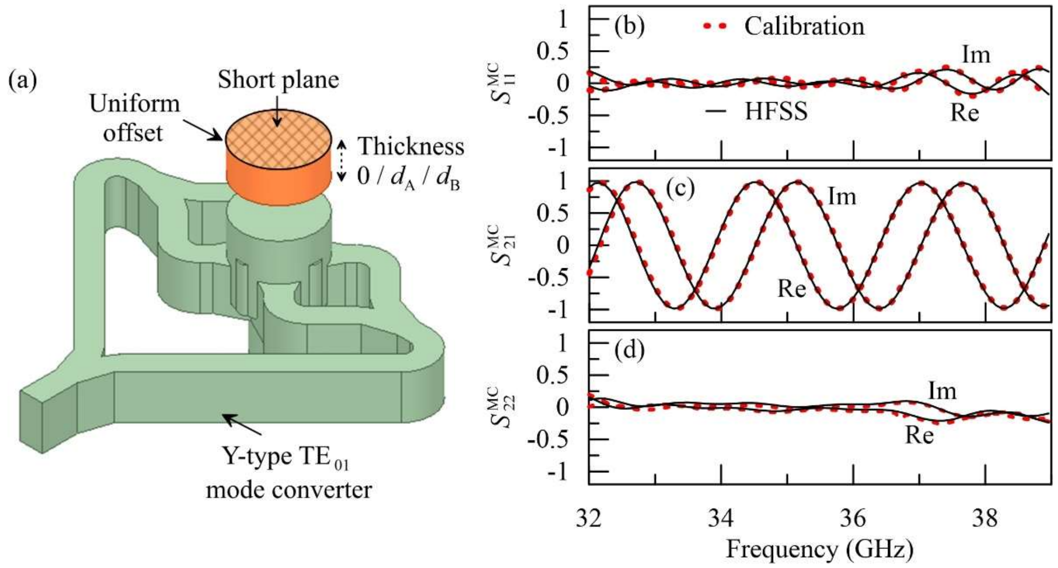

Fig. S2 (a) Offset scheme for the calibration of the mode converter. The color regions show the waveguide tunnels. Uniform offset waveguide has the same radius of the output probe of the mode converter and its end is sealed by the metal plate (short). (b) S_{11}^{MC}. (c) $S_{12}^{\mathrm{MC}} = S_{21}^{\mathrm{MC}}$ and (d) S_{22}^{MC}.

Based on Eqs. (S4) and (S5), it is possible to extract the reduced scattering matrix of the TE_{01} mode converter with the three individually measured reflection coefficients S_{11}^{S}, S_{11}^{OA}, and S_{11}^{OB}.

The overall reflection coefficient measured by the mode converter probing the sample under test ($\bar{R}$) is

$$\bar{R} = S_{11}^{\mathrm{MC}} + \frac{S_{12}^{\mathrm{MC}} S_{21}^{\mathrm{MC}} R}{1 - S_{22}^{\mathrm{MC}} R}. \tag{S6}$$

Note that $\bar{R}$ is the actual measurable reflection coefficient instead of R derived in Eq. (4). Substituting Eq. (4) into Eq. (S6), we have

$$R = \frac{\bar{R} - S_{11}^{\mathrm{MC}}}{\bar{R} S_{22}^{\mathrm{MC}} - S_{11}^{\mathrm{MC}} S_{22}^{\mathrm{MC}} + S_{12}^{\mathrm{MC}} S_{21}^{\mathrm{MC}}} = \frac{-\left(k_{z0} - k_{zs}\right) e^{-2ik_{zs}d} + \left(k_{z0} + k_{zs}\right)}{-\left(k_{z0} + k_{zs}\right) e^{-2ik_{zs}d} + \left(k_{z0} - k_{zs}\right)}. \tag{S7}$$

Equation (S7) serves as the final equation for numerical root searching of the complex permittivity of ε_s embedded in k_{zs}.
